# Host genetic backgrounds: the key to determining parasite-host adaptation

**DOI:** 10.3389/fcimb.2023.1228206

**Published:** 2023-08-10

**Authors:** Caixia Ye, Lianhua Zhang, Lili Tang, Yongjun Duan, Ji Liu, Hongli Zhou

**Affiliations:** ^1^ Clinical Medical Research Center, The Second Affiliated Hospital, Army Medical University, Chongqing, China; ^2^ Department of Pediatrics, Yunyang Women and Children’s Hospital (Yunyang Maternal and Child Health Hospital), Chongqing, China; ^3^ Department of Surgery, Yunyang Women and Children’s Hospital (Yunyang Maternal and Child Health Hospital), Chongqing, China; ^4^ The 3rd Affiliated Teaching Hospital of Xinjiang Medical University (Affiliated Tumor Hospital), Urumqi, China; ^5^ Sun Yat-sen University Cancer Center, State Key Laboratory of Oncology in South China and Collaborative Innovation Center for Cancer Medicine, Guangzhou, China

**Keywords:** parasite, host, adaptation, genetic background, immune response

## Abstract

Parasitic diseases pose a significant threat to global public health, particularly in developing countries. Host genetic factors play a crucial role in determining susceptibility and resistance to infection. Recent advances in molecular and biological technologies have enabled significant breakthroughs in understanding the impact of host genes on parasite adaptation. In this comprehensive review, we analyze the host genetic factors that influence parasite adaptation, including hormones, nitric oxide, immune cells, cytokine gene polymorphisms, parasite-specific receptors, and metabolites. We also establish an interactive network to better illustrate the complex relationship between host genetic factors and parasite-host adaptation. Additionally, we discuss future directions and collaborative research priorities in the parasite-host adaptation field, including investigating the impact of host genes on the microbiome, developing more sophisticated models, identifying and characterizing parasite-specific receptors, utilizing patient-derived sera as diagnostic and therapeutic tools, and developing novel treatments and management strategies targeting specific host genetic factors. This review highlights the need for a comprehensive and systematic approach to investigating the underlying mechanisms of parasite-host adaptation, which requires interdisciplinary collaborations among biologists, geneticists, immunologists, and clinicians. By deepening our understanding of the complex interactions between host genetics and parasite adaptation, we can develop more effective and targeted interventions to prevent and treat parasitic diseases. Overall, this review provides a valuable resource for researchers and clinicians working in the parasitology field and offers insights into the future directions of this critical research area.

## 1 Introduction

Parasitic diseases are important among human infectious diseases. The prevalence of parasitic infection in tropical and subtropical regions is more severe owing to humidity and relatively lagging economic and sanitary conditions (Dejon-Agobe et al., 2022; Kalkman et al., 2022; Taylor et al., 2022); thus, parasitic infection remains a major public health problem in these regions. During the course of biological evolution, intricate relationships have been formed between organisms, among which the term symbiosis describes the phenomenon that two different organisms live together. Symbiosis can be further divided into commensalism, mutualism and parasitism. Parasitism means that two organisms live together, one of which, the parasite, benefits and the other, the host, suffers. Parasitism originates from contact between parasites and hosts. Once a host is infected, parasites trigger the defensive response of the host, but with different outcomes. First, hosts can expel all of the worms and acquire the ability to resist reinfection; second, hosts can eliminate only some worms, which is the case upon which most relationships between hosts and parasites are based; and third, hosts can fail to control the parasites effectively, leading to sustained parasite survival, the development of parasitism, and thus evident pathological changes, clinical symptoms and even death (Micucci et al., 2010; Lotter et al., 2013; Zhou et al., 2021). The outcomes of parasite-host adaptation are generally closely related to host genetic backgrounds, yet summaries in this field are lacking. In this article, the host adaptation of parasites and the potential mechanisms remarkably influenced by host genetic backgrounds are reviewed (Shen et al., 2020). It is expected that this review will provide new insights for the in-depth understanding of parasite-host adaptation as well as strategies to prevent and control parasitic infection in the future.

## 2 Main text

### 2.1 Literature search strategy

In this study, we reviewed scientific studies with no time restriction to identify studies focusing on the factors of host genetic backgrounds that affect parasite-host adaptation. A total of 19 parasites whose adaptation has been documented were included in this study, including protozoans, flagellates, sporozoans, trematodes, cestodes and nematodes. Search terms were the combinations of “Latin name for parasite”, “host”, “host susceptibility”, “host resistance”, and “host adaptation” using the online databases PubMed (https://www.ncbi.nlm.nih.gov/pubmed/) or Web of Science (http://apps.webofknowledge.com/UA_GeneralSearch_input.do?product=UA&search_mode=GeneralSearch&SID=5ChWtwlripdAqJRyqsq&preferencesSaved=). After removing duplicated articles, we screened the titles and abstracts of the articles according to the following inclusion criteria: (1) studies published in English and (2) articles describing the differences among parasite migration, development, survival and pathogenicity caused by host genetic backgrounds. The exclusion criteria were as follows: (1) studies that were duplicates of graduate theses; (2) studies of influencing factors with no significant differences in hosts of different genetic backgrounds; and (3) studies not describing or related to parasite-host adaptation.

### 2.2 Protozoan

#### 2.2.1 Entamoeba histolytica

*Entamoeba histolytica* (*E. histolytica*) is the only intestinal amebiasis known to cause amebiasis in humans, characterized by intestinal damage and amoebic liver abscess. Humans are permissive hosts for *E. histolytica*. Monkeys, cats, dogs, and mice can also serve as incidental hosts but retain natural resistance to amoebae. Some rodent strains, such as C3H/Hej, CBA/J, Mongolian gerbils, and hamsters, develop amoebic liver abscesses similar to humans, while others, such as BALB/c, C57BL/6 and rats, are somewhat resistant to the development of amoebic liver abscesses, which is attributed to the increasing levels of nitric oxide (NO) and some higher levels of cytokines, such as IL-10 (Chadee and Meerovitch, 1984; Ivory et al., 2007; Olivos-Garcia et al., 2018). Mucin deficiency leading to the failure of mucin to cleave the amoebic caspase in some hosts might cause natural resistance. Studies have also confirmed that IL-10 deficiency reduces the synthesis of host colonic mucin MUC2. Therefore, IL-10-deficient mice with defective mucin synthesis are susceptible to amebic invasion of the epithelium (Ivory et al., 2007). In contrast, rats are completely resistant to amebiasis by virtue of the elimination of amebiasis through serum complement components at an early stage (the first 6 hours after infection) (Olivos-Garcia et al., 2018). In addition, the expression of inducible nitric oxide synthase (iNOS) stimulated by amoebae varies widely among the macrophages of different hosts. For example, iNOS is highly expressed by alveolar macrophages obtained from rats, but not in those obtained hamsters or humans, when stimulated by LPS or IFN-γ, thus participating in the elimination of amoeba (Ramirez-Emiliano et al., 2007). In the human population, amoeba infection is more common in men than in women (Groneberg et al., 2022). Similarly, *E. histolytica* activates a stronger immunopathological response and results in larger liver abscesses in male mice than in female mice. Male HIF-1α knockout (KO) mice show no difference from female mice in the size of amoebic liver abscesses as a consequence of the decrease in Th17 cells and cytokine IL-6 located downstream (Groneberg et al., 2022). Hence, HIF-1α-regulated Th17 cells and cytokines might be important factors determining the differences in immunopathology between males and females. Additionally, the host status of hormones, such as testosterone and leptin, may affect the resistance of the host to amebiasis. Testosterone shows an inhibitory effect on host resistance by suppressing IFN-γ secretion by natural killer T cells, while leptin enhances this secretion (Duggal et al., 2011; Lotter et al., 2013). Furthermore, as reported, MHC polymorphisms such as HLA-DRB1*03:0101 and HLA-DRB1*11:0101 in humans may be associated with higher susceptibility to amebiasis (Al-Khaliq and Mahdi, 2018). Altogether, these findings imply that host genetic background-related factors such as NO, IL-10, complement, hormone and MHC polymorphisms are involved in parasite-host adaptation.

### 2.3 Flagellates

#### 2.3.1 Leishmania

Leishmaniosis is a zoonosis caused by *Leishmania* that is transmitted through the bite of female *phlebotomine sandflies* carrying the promastigote in their salivary glands. These parasites infect not only humans but also other vertebrates, including rodents, lagomorphs and mustelids (Villanueva-Saz et al., 2022). *Leishmania braziliensis* (*L. braziliensis*) is the agent of cutaneous leishmaniasis (CL), the most prevalent clinical form of leishmaniasis in Brazil. However, only a few infected people exhibit symptoms. The underlying mechanism varies but is genetically correlated. First, a single nucleotide polymorphism (SNP) is involved. As reported, people carrying the rs2275913 A SNP of the IL17A gene are more vulnerable to *L. braziliensis* infection than those carrying the rs2275913 G SNP (Goncalves de Albuquerque et al., 2019). For carriers of the rs16944 T/C SNP of the IL1B gene, those with the C/C genotype are more susceptible to CL (da Silva et al., 2019). The effects of cytokines on susceptibility may be opposite in mice with different genetic backgrounds. It was reported that IL-18 knockout 129×CD1 and DBA/1 genetic background mice showed higher susceptibility to *Leishmania* infection than wild-type (WT) mice, likely by suppressing the Th1 response. Conversely, the enforced Th1 response led the IL-18 knockout BALB/c background mice to exhibit much resistance to Leishmania infection(Wei et al., 2004) and only BALB/c background mice shows the response specific to IL-18 knockout while other background mice like 129×CD1, DBA/1 and C57BL/6(Clay et al., 2014) exhibit increased susceptibility to Leishmania infection. In addition, the resistance of hosts is also related to other cytokines, such as IL-1α, IL-10, IL-22 and IL-32(Schamber-Reis et al., 2013; Dos Santos et al., 2020). IL-1α is required for optimal IL-12-induced Th1 development in BALB/c but not C57BL/6 mice (Shibuya et al., 1998). The less IL-1α in response to L. major from BALB/c compared with C57BL/6 mice, providing partial explanation for the disparate outcomes of infection between these two mouse strains (Von Stebut et al., 2003). IL-22 is a member of the IL-10 family cytokines. The increased expression of the IL-10 was significantly associated with enhanced sensitivity to L. major infection in UNC93B1 mutant mice for the reason of the skewed expression pattern towards upregulation of IL-10 (Schamber-Reis et al., 2013). The genetic background of IL-32 variation in host can influence its susceptibility to Leishmania (Dos Santos et al., 2020). The IL32 rs4786370 genetic variation was associated with protection against ATL, while IL32 rs4349147 susceptibility to localized cutaneous and mucosal leishmaniasis.

#### 2.3.2 Trypanosoma

Infectious *Trypanosoma* in humans includes *Trypanosoma brucei gambiense* (*T. b. gambiense*), *Trypanosoma brucei rhodesiense* (*T. b. rhodesiense*) and *Trypanosoma cruzi* (*T. cruzi*). *T. b. gambiense* and *T. b. rhodesiense* are prevalent in Africa and are transmitted by the *Glossina palpalis* and *Glossina tsetse* flies, respectively. The reservoirs of *T. b. gambiense* include humans, cattle, pigs, sheep and dogs, whereas those of *T. b. rhodesiense* comprise humans, sheep, cattle, lions and dogs (Alfituri et al., 2020). For the American subspecies, *T. cruzi* transmitted through the bite of the triatomine bug is virulent to humans and other mammals, including foxes, squirrels, anteaters, dogs and cats (Rosypal et al., 2014; Busselman and Hamer, 2022). Because *T. cruzi* is the most intensively studied *Trypanosoma* species, we mainly summarize the host adaptation of *T. cruzi*.

Genetic backgrounds play important roles in the susceptibility of hosts to *T. cruzi*. Mice with a BALB/c background are resistant to *T. cruzi*, while C57BL/6 mice are susceptible to *T. cruzi*. In detail, different mouse strains have distinct major histocompatibility complex (MHC) haplotypes, which affect their antigen presentation and recognition. As is known, C57BL/6 mice have the H-2b haplotype, which is more efficient in presenting peptides derived from intracellular pathogens, whereas BALB/c mice have the H-2d haplotype, which is more efficient in presenting peptides derived from extracellular pathogens. And this genetic background difference results in their different susceptibility to *T. cruzi*. Moreover, In B10 or DBA/1 mice, the H-2q haplotype is related to resistance, whereas the C3H genetic background is related to susceptibility (Powell and Wassom, 1993). It is reported that C57BL/6 mice produced higher levels of IFN-γ and lower levels of IL-4 than BALB/c mice, indicating a Th1 immune response(Bergersen et al., 2021). However, C57BL/6 shows more susceptible to *T. cruzi* than BALB/c mice, suggesting Th2 immunity likely plays a more important role in the elimination of *T. cruzi*. The polymorphism of cytokine genes, such as the IL-1, IL-4, IL-13, IL-17, IL-18, TNF-α and IFN-γ genes, may impact their expression and lead to diverse immune responses, resulting in different parasite adaptation and host susceptibility (Hiyama et al., 2001; Miyazaki et al., 2010; Chessler et al., 2011; Abad Dar and Holscher, 2018; Strauss et al., 2019). For example, the amplification of the IL-13 gene in mice increases the susceptibility to *T. cruzi*, elevates parasite parasitemia and impairs survival because of the enhanced activation of arginase, which promotes parasite replication (Abad Dar and Holscher, 2018). As demonstrated, arginase limits the production of NO and increases the level of polyamine (Abad Dar and Holscher, 2018), and NO enhances the resistance of the host to parasites, but polyamine provides materials for replication. Consistently, higher NO levels were observed in C57BL/6 mice during acute infection with *T. cruzi*, and iNOS KO mice showed a higher susceptibility to *T. cruzi* (Holscher et al., 1998). Furthermore, insufficient NO causes a higher susceptibility to *T. cruzi* in BALB/c mice than in XID mice, which is negatively related to excessive IL-10 secreted by B-1 cells (da Rocha et al., 2019). In addition, parasite-specific IgG and sex differences are also involved in parasite-host adaptation, but further investigation of the underlying mechanisms is needed (Perez et al., 2005; Micucci et al., 2010).

#### 2.3.3 Trichomonas vaginalis

*Trichomonas vaginalis* (*T. vaginalis*) is a parasite infecting the human vagina and urinary tract, where it causes inflammation. Hitherto, it has been believed that *T. vaginalis* causes disease only in humans (Corbeil, 1995). Both men and women are susceptible to *T. vaginalis*, and infected individuals can be either symptomatic or asymptomatic. Males display stronger resistance to *T. vaginalis*, which might be attributed to pre-T cells, while estrogen helps *T. vaginalis* adhesion in females (Martinotti et al., 1988). Symptomatic patients show higher CP30 than asymptomatic patients among females (Yadav et al., 2007), whether it is the same among males remains further exploration. Furthermore, the elevation of IgM, IgG1 and IgG2b in patient urine samples was found to be related to more severe symptoms (Imam et al., 2007). In addition, the virulence of *T. vaginalis* could be modulated by MHC, the sensitivity of T cells and the activation of phagocytes in hosts (Caterina et al., 1996). A mouse strain harboring H-2^d^ was shown to be susceptible to infection with *T. vaginalis*, but strains with H-2^k^ were resistant (Caterina et al., 1996). Notably, the differences due to hormones, immunoglobulin and MHC depend on genetic divergence, which would affect the susceptibility of hosts. However, the direct evidences supporting genetic divergence-determined susceptibility remain more further investigations.

### 2.4 Sporozoan

#### 2.4.1 Plasmodium

*Plasmodium* has a wide range of hosts, including humans, birds, reptiles, apes, monkeys, rodents, ungulates and other kinds of animals (Perkins, 2014; Templeton et al., 2016; Galen et al., 2018; Matta et al., 2018; Valkiunas et al., 2018). Currently, six species of *Plasmodium* have been proven to cause malaria in humans, namely, *Plasmodium. falciparum* (Pf), *Plasmodium. vivax* (Pv), *Plasmodium. knowlesi* (Pk), *Plasmodium. malariae* (Pm), and two subspecies of *Plasmodium ovale* (Po) (Sutherland et al., 2010). Among the *Plasmodium* species infectious to humans, Pf belongs to the subgenus *Laverania*, for which host selection is extremely strict. Pf infects only humans, whereas the others specifically infect apes such as chimpanzees or gorillas, which is determined by the variance in receptor-ligand binding during the adsorption of *Plasmodium* to erythrocytes, as widely recognized (Liu et al., 2016). Although genomics between species within the subgenus *Laverania* is relatively conserved, the genes encoding erythrocyte adsorption ligands, such as those of the Stero gene family and Rh gene family, are quite different (Otto et al., 2018; Plenderleith et al., 2018). Furthermore, EBA165 in the erythrocyte binding-like gene family is silenced in the Pf genome but expressed in other *Laverania* species. When Pf expresses EBA165, Pf gains the ability to infect apes rather than infect humans (Proto et al., 2019). On the other hand, the surface receptors of human and ape erythrocytes are distinct. Studies have shown that the Rh5 molecule of Pf is the only ligand present on all Pf strains that can bind to basigin (BSG, CD147) on the surface of erythrocytes (Crosnier et al., 2011). The affinity of Rh5 to human BSG is significantly higher than that of chimpanzees and gorillas for two key BSG sites, F27 and K191, thereby increasing the affinity for Rh5 (Forni et al., 2015). In addition, differences in the sialic acids on the surface of erythrocytes might affect the infection outcomes for humans and apes. In apes, Neu5Gc regulates the production of sialic acid. Neu5Ac cannot be converted into Neu5Gc in humans due to the lack of cytidine monophosphate N-acetylneuraminic acid hydroxylase, thus showing the differences in plasmodium adsorption to erythrocytes in different species (Muchmore et al., 1998; Hayakawa et al., 2001).

Hosts of *Plasmodium* can be divided into hosts with intermediate host adaptation and hosts with definitive host adaptation. Humans are a special intermediate host, which is mainly represented in the fact that humans in some populations are naturally resistant to malaria. The ligand-receptor pair composed of Duffy-binding protein (DBP-RII) on the surface of Pv and Duffy antigen receptor chemokine (DARC) on the surface of erythrocytes is an essential molecular basis for infection. The surfaces of the erythrocytes of most black people in West Africa lack DARC because of natural selection, so these individuals are naturally resistant to Pv (Chitnis and Sharma, 2008; de Sousa et al., 2014; Payne et al., 2017). Abnormal structures of erythrocytes, including those associated with the sickle cell (HbAS) and HbAC (HbAC) traits, could result in inhibitory effects on Pf, thus enhancing resistance in people throughout Africa harboring these two structures. However, the specific mechanism has not been fully elucidated (Kreuels et al., 2010; Goncalves et al., 2016). In addition, G6PD is essential to maintain the growth and development of *Plasmodium* (Usanga and Luzzatto, 1985). Therefore, populations with G6PD deficiency generally display enforced resistance to *Plasmodium* (Louicharoen et al., 2009). Definitive hosts of *Plasmodium* include mosquitoes, *Hylemyia* and midges (Hellgren et al., 2008; Martinez-de la Puente et al., 2011), and mosquitoes are the main definitive host, while only *Anopheles* mosquitoes can transmit *Plasmodium* as a result of ookinete immune evasion (Knockel et al., 2013). At the molecular mechanistic level, Pfs47 on the surface of Pf binding to P47Rec is specifically expressed in the stomach of *Anopheles* mosquitoes and mediates the immune escape of ookinetes, which is further confirmed by the blunted Pf infection upon P47Rec silencing (Molina-Cruz et al., 2020).

#### 2.4.2 Toxoplasma gondii

*Toxoplasma gondii* (*T. gondii*) is an obligate intracellular and opportunistic parasite that parasitizes all karyocytes and mainly spreads by peroral infection and vertical transmission. The life history of *T. gondii* is divided into the sexual stage (enteral phase) and the asexual stage (parenteral period). Only cats and felines are definitive hosts for *T. gondii* (Frenkel et al., 1970) because the lack of ω-6-desaturase in cat intestinal epithelial cells leads to a high content of linoleic acid, which indicates that ω-6-desaturase can foster host adaptation by *T. gondii*. Consistently, the ω-6-desaturase inhibitor or a high linoleic acid diet was found to enable *T. gondii* to develop to the sexual stage in mice (Martorelli Di Genova et al., 2019).

The asexual part of *T. gondii* exhibits a flexible form of host selection, with hosts including all warm-blooded animals such as mice, rabbits, sheep, cattle and some cold-blooded animals (Dubey, 2008). As reported, the host adaptation of *T. gondii* depends on immune factors, NO and reactive oxygen species (ROS). The higher level of NO in rats is more resistant to *T. gondii* when than that in mice (Witola et al., 2017). However, recent studies have shown that the knockout of the iNOS gene enables stronger resistance against *T. gondii* in rats, which is possibly because the loss of iNOS frees the ROS system of the mononuclear phagocyte system. ROS have been well documented to participate in the elimination of pathogens; hence, the targeting of ROS is likely an effective strategy to kill *T. gondii* and to avoid the spread of this pathogen in rats (Wang et al., 2021).

#### 2.4.3 Cryptosporidium

*Cryptosporidium* is an opportunistic intracellular parasite that parasitizes intestinal epithelial cells, and its life history consists of sexual and asexual generations. Upon infection, *Cryptosporidium* mainly causes gastrointestinal symptoms such as diarrhea. *Cryptosporidium* and its diverse subspecies can infect almost all organisms, including humans, cattle, sheep, pigs and other species (Xiao and Fayer, 2008). Infectious *Cryptosporidium* species to humans mainly include *Cryptosporidium parvum* (*C. parvum*) and *Cryptosporidium hominis* (*C. hominis*). In addition, several other *Cryptosporidium* species also infect patients with immunodeficiency diseases such as acquired immunodeficiency syndrome (AIDS) (Hunter and Nichols, 2002; Ahmadpour et al., 2020). As an opportunistic parasite, *Cryptosporidium* infection is closely related to an immunocompromised or immunodeficient status, and hosts display different susceptibilities according to HLAI/II types (Kirkpatrick et al., 2008). A high infection rate of *Cryptosporidium* is influenced by HLA DQB1*0301 and HLA B*15. In fact, infants are more susceptible to *Cryptosporidium* than adults (Hunter et al., 2004). Moreover, patients with immune deficiency related to T cells exhibit higher susceptibility to *Cryptosporidium*, which indicates that T-cell-mediated adaptive immunity probably plays a major role in parasite-host adaptation (Winfield, 1997). In the case of Cryptosporidium infection, T-cell-mediated adaptive immunity is believed to be important for host resistance (McDonald, 2000; McNair and Mead, 2013; Russler-Germain et al., 2021). The genetic background of the host can influence the T-cell response and subsequent parasite adaptation. Genetic variations in immune-related genes, including those involved in T-cell activation, cytokine production, and antigen presentation, can impact the effectiveness of the T-cell response against Cryptosporidium. For example, polymorphisms in genes (like SNPs of LTα, IFNβ1 and TNFα) can affect the production and function of cytokines, which in turn can influence the balance between Th1 and Th2 responses (Davhana et al., 2020; Kloch et al., 2023). However, a dearth of experimental studies at the genetic level exists for the majority of factors that have been unequivocally identified as influencing parasite host adaptation. Nevertheless, it is possible to engage in speculation regarding the genetic underpinnings of these influential factors and elucidate the association between the host gene corresponding to the factor and host adaptation. In contrast, B cells show no significant effect on host resistance to *Cryptosporidium* (Gomez Morales et al., 1996).

### 2.5 Trematodes

#### 2.5.1 Clonorchis sinensis

The hosts of *C. sinensis* comprise the first intermediate host (freshwater snails), second intermediate hosts (freshwater fish and shrimp) and definitive hosts. More than 30 mammals, including humans, serve as definitive hosts (Li et al., 2018). Humans are generally susceptible to *C. sinensis* regardless of sex, age, or race. After infection, *C. sinensis* metacercariae migrate to and parasitize human intrahepatic bile ducts, where they develop into adults and lay eggs under the chemotaxis of cholic acid, suggesting that cholic acid affects the host adaptation of *C. sinensis* (Li et al., 2018). Furthermore, the susceptibility of hosts to *C. sinensis* is correlated with host immunity. C3H/HeN mice with higher levels of IgE, IFN-γ, IL-13 and Th1/Th2 immune responses are less susceptible than C57BL/6, BALB/C and ICR mice (Lee et al., 2006; Uddin et al., 2012). However, the development of *C. sinensis* is impaired in immunocompromised SCID and nude mice, which implies that immune factors probably exert dual functions in mouse strains with different genetic backgrounds (Yoon et al., 2001).

#### 2.5.2 Fasciola hepatica

The hosts of *Fasciola hepatica* (*F. hepatica*) comprise the intermediate host (Lymnaeidae) and definitive hosts (diverse mammals, including humans). Differences in genetic background among intermediate hosts have notable influences on susceptibility to *F. hepatica* (Cruz-Reyes and Malek, 1987). Lymnaeidae includes *Pseudosuccinea columella* and *Fossaria cubensis. Pseudosuccinea columella* is more susceptible to *F. hepatica* than *Fossaria cubensis*, which is possibly the cause of the divergence in genetic background and size between snails (Cruz-Reyes and Malek, 1987). *F. hepatica* is a common parasite that parasitizes the intrahepatic bile ducts of the definitive host, including common hosts (cattle and sheep) and uncommon hosts (rodents, rabbits and humans). The adaptation of *F. hepatica* varies in different definitive hosts. In uncommon hosts, the egg size is smaller than that in common hosts, and the ability of eggs to develop into miracidia is weaker, thus leading to a reduction in their prevalence (Abrous et al., 1998). In addition, the resistance of definitive hosts to *F. hepatica* is closely associated with the adaptive immune response. The thymus, the major reservoir of T cells, is the contributing factor of host resistance to *F. hepatica*. As reported, infection with *F. hepatica* is lethal to congenitally athymic nude mice (Eriksen, 1980). Furthermore, the Th1 immune response promotes the elimination of hosts by *F. hepatica* (Pleasance et al., 2011). Consistently, indigenous thin-tailed sheep with early and locally occurring Th1 immune responses were found to exhibit stronger resistance to *F. hepatica* infection (Pleasance et al., 2011).

#### 2.5.3 Schistosoma

The hosts of *Schistosoma* include intermediate and definitive hosts. The intermediate hosts comprise *Oncomelania hupensis*, *Biomphalaria*, *Bulinus*, *Tricula aperta* and the small *Robert snail*, while the definitive hosts include humans, cattle, pigs, dogs, sheep, rodents, monkeys and baboons. Based on the differences in resistance to *Schistosoma*, definitive hosts are classified into nonpermissive hosts and permissive hosts. Nonpermissive hosts with stronger resistance to *Schistosoma* include *Microtus fortis*, rats and buffalo (Yang et al., 2013; Shen et al., 2020), in which *Schistosoma* fails to develop to sexual maturity. Permissive hosts exhibiting less resistance to *Schistosoma* include mice, goats and humans, where the adult worms lay eggs and consequently develop hepatic lesions characterized by egg granulomas (Yang et al., 2013).

The intermediate hosts of *S. mansoni* mainly include the NMRI and BS-90 strains of *B. glabrata*. The NMRI strain is susceptible to *S. mansoni*, whereas the BS-90 strain is not (Knight et al., 1999). Resistance and susceptibility are closely related to some special genes. At the genome level, these genes are in the same linkage group with a short distance from each other, of which the genes including Prim1_910, Prim1_771, Prim6_1024 and Prim7_823 are related to resistance, while Prim24_524 is associated with susceptibility (Ittiprasert et al., 2013). At the phenotype level, the resistance of BS-90 strains to *S. mansoni* could be reversed by knocking down the fibrinogen-related protein FREP3 (Zhang et al., 2008; Hanington et al., 2012). The susceptibility of *B. glabrata* is also closely linked to the expression of HSP70 and HSP90 induced by mild heat shock (32 °C), leading to the stronger susceptibility of BS-90 strains to *S. mansoni*. In addition, the inhibition of HSP 90 reverses the susceptible phenotype of NMRI strains (Ittiprasert and Knight, 2012). Furthermore, *Biomphalaria alexandrina* serves as another intermediate host of *S. mansoni* with higher resistance that is likely attributable to SOD1 activity, levels of polymorphic mucin (SmPoMuc) and NO, determined by the genetic background (Bender et al., 2007). Environmental light can also modulate the susceptibility of *B. glabrata* to *S. mansoni*, but the underlying mechanisms remain elusive (Steinauer and Bonner, 2012).

The resistance and susceptibility phenotypes of definitive hosts are closely linked to immune factors, parasite-induced gene expression and hormone levels determined by the genetic background. Immune factors include humoral immunity components, cytokines and immune cells. At the humoral immunity level, the resistance of *M. fortis* to *S. japonicum* is dependent on serum albumin and IgG3 helping to eliminate worms (Jiang et al., 2010; Li et al., 2013), whereas in rats, this resistance depends on IgG2a and IgE (Shen et al., 2020). The application of purified albumin from *M. fortis* infecting mice exerts significant effects on worm load reduction, suggesting a promising target for therapy (Jiang et al., 2010; Li et al., 2013). In terms of cytokines, diverse inflammatory factors (including FN-γ, IL-1β, GM-CSF, M-CSF, MCP-1, VEGF, IL-2, IL-3, IL-4, IL-5, IL-10, IL-12, IL-13 and IL-17) are remarkedly upregulated in hosts upon infection (Hu et al., 2017). Further blockade of IL-4 and IL-13 was shown to notably elevate the worm burden of *S. mansoni* in rats, indicating that Th2-type cytokines enhance the resistance of nonpermissive hosts (Khalife et al., 2000). However, in permissive hosts, Th2 responses seem beneficial to parasites (Khalife et al., 2000). Regarding immune cells, eosinophils, macrophages and lymphocytes function to eliminate worms in *M. fortis* (Shen et al., 2020). Based on these findings, adoptive immunotherapy with bone marrow transplanted from *M. fortis* significantly inhibited the development of worms and reduced the number of worm eggs in immunocompromised mice, showing promising therapeutic efficiency. At the parasite-induced gene expression level, microRNAs, including miR-705 and miR-122, inhibit the development of *S. japonicum* by affecting lipid uptake, and high levels of iNOS in rats inhibit the development of *S. japonicum* (Shen et al., 2017). Significantly, the proteins Mf-HSP90a (Gong et al., 2010), Mf-KPNA2 (Cheng et al., 2011), and Mf-E77.43 (Wang et al., 2016) in *M. fortis* (Shen et al., 2017) and the serum in rats can directly kill *S. japonicum* (Wang et al., 2005), providing a candidate strategy for schistosomiasis treatment. Furthermore, the differences in hormone levels between males and females also affect the development of *S. mansoni*, as observed for testosterone in males, which enhances resistance and reduces the adult worm burden in hosts (Nakazawa et al., 1997).

#### 2.5.4 Cestodes

##### 2.5.4.1 Hymenolepis nana and Hymenolepis diminuta

The life cycle of *Hymenolepis nana* (*H. nana*) is analogous to that of *Hymenolepis diminuta* (*H. diminuta*). Mice and humans serve as their definitive hosts, in which adult worms parasitize the intestine. Similar to most parasites, NO presents obvious inhibition capacity to *hymenolepis* (Mahmoud and Habib, 2003). In addition, immune factors are also involved in the host adaptation of this parasite. As reported, thymectomized mice displayed a slower efficiency of worm elimination for hymenolepis (Andreassen et al., 1978), suggesting that the resistance of hosts presents certain immune-dependent characteristics (Palmas et al., 1993). In addition, cytokines (such as IL-5 and IL-13) and the inflammation-related transcription factor stat6 promote the expulsion of *H. diminuta* (McKay and Khan, 2003; Ryan and Oghumu, 2019). Moreover, immune cells, including mast cells and neutrophils, enforce the resistance of hosts to this parasite (Gonzalez et al., 2018).

##### 2.5.4.2 Echinococcus granulosus

The hosts of *Echinococcus granulosus (E. granulosus)* comprise intermediate hosts and definitive hosts. Artiodactyls (such as sheep, cattle, camels, pigs and deer), horses, kangaroos, rodents, primates and humans are intermediate hosts that can be infected by the hydatid cyst of *E. granulosus* and develop echinococcosis (Maillard et al., 2009; Otero-Abad and Torgerson, 2013; Kagendo et al., 2014). Dogs, wolves and jackals serve as the definitive hosts parasitized by the adult worms of *E. granulosus*, in which eggs are produced (Kagendo et al., 2014). *Mongolian gerbils*, *Meriones Meridianus*, and *Lagurus* act as alternative definitive hosts, where the development of worms is distinct from that in natural definitive hosts (Oku et al., 2002; Conchedda et al., 2006).

Regarding intermediate hosts, most infections with the hydatid cysts of *E. granulosus* result in multiorgan involvement. Specifically, in rodents, liver involvement is most common as a result of the existence of Kupffer cell-specific receptor (KCR), to which hydatid cyst laminated layer glycans directly bind (Fadden et al., 2003; Diaz et al., 2015). In contrast, large mammals, especially ruminants, mainly manifest extrahepatic organ involvement, which may be related to the larger diameter of lymphatic vessels in jejunal villi than that in nonruminant animals, leading to *Echinococcus* being more likely carried in lymph rather than in the portal circulation (Heath, 1971; Thompson, 2017). The immune factors established by the distinct genetic backgrounds of hosts result in distinct resistance to *E. granulosus*. When compared with BALB/c mice, C57BL/6 mice present lower vulnerability, which is attributed to the H-2-IAb haplotype T-cell epitope-mediated stronger immune responses to parasites (Miles et al., 2020; Garcia-Luna et al., 2021). Serum from C57BL/6 mice showed explicit therapeutic efficiency in BALB/c mice infected with *E. granulosus*, which is caused by IgG1 (Mourglia-Ettlin et al., 2016a). The pathogen associated with pattern receptor TLR4 also affects parasite-host adaptation. As reported, TLR4 SNPs and the heterozygous mutant Asp299Gly (A/G) genotype were closely associated with susceptibility to *E. granulosus* (Noori et al., 2018). In general, the Th1 response displays protective function against hydatid cysts, and the Th2 response shows a positive correlation with susceptibility (Siracusano et al., 2012). However, several critical cytokines (IL-4 and IL-10) of the Th2 response have opposite killing effects on protoscoleces by elevating the levels of NO, which enhances the cytotoxic effect in C57BL/6 mice upon infection (Amri et al., 2009; Mourglia-Ettlin et al., 2016b). In addition, the sex and age of the hosts are also closely related to worm resistance (Blancas Mosqueda et al., 2007; Mourglia-Ettlin et al., 2018).

##### 2.5.4.3 Echinococcus multilocularis

The life cycle of *E. multilocularis* is similar to that of *E. granulosus*. The intermediate hosts of *E. multilocularis* include rodents, cattle, sheep and humans, where the worms fail to develop into sexual maturation (Weingartner et al., 2022). Animals such as foxes, dogs, wolves, badgers and cats serve as definitive hosts, in which adult worms lay eggs that are excreted with feces (Hansen et al., 2004; Karamon et al., 2019).

In general, *E. multilocularis* parasitizes the host liver in the form of alveolar cercariae and occasionally in other tissues, such as the brain and lungs (Maamri et al., 2022), since insulin in the liver can promote the development of *E. multilocularis* through the evolutionarily conserved insulin signaling pathway of worms (Hemer et al., 2014). The resistance of different rodents to *E. multilocularis* is not identical as a consequence of the distinct parasite-specific humoral and cellular immunity gradually established in various strains. Some rodent strains, for example, DBA/2 mice, show higher susceptibility to alveolar echinococcosis than C57BL/6 mice, whereas humans are generally more resistant than nonhuman primates (Nakao et al., 2011; Armua-Fernandez et al., 2016). In addition, other immune factors, including neutrophils and MHC polymorphisms, lower the susceptibility to and onset of echinococcosis in the hosts (Vuitton et al., 2006; Joekel et al., 2021). Hosts with HLA-DR3^+^ and HLA-DQ2^+^ haplotypes are more susceptible to progressive alveolar echinococcosis. In addition, the lack of iNOS enforces the resistance of mice, which is contradictory to most prior reports, and dexamethasone affects susceptibility by suppressing the immune response (Dai et al., 2003; Armua-Fernandez et al., 2016).

### 2.6 Nematodes

#### 2.6.1 Trichuris trichiura Linnaeus

As the host of *T. trichiura*, humans are the only source of infection in which adult worms parasitize the cecum and lay eggs that are excreted into the natural environment and develop to the infectious stage in soil with suitable humidity and temperature (Bethony et al., 2006). After invasion, infective eggs migrate through the small intestine and intestinal lumen, eventually arrive at the cecum, develop into adult worms and cause trichuriasis in hosts (Bethony et al., 2006). Mouse models are the animal models most commonly exploited for trichuriasis investigation, and the divergent genetic backgrounds of mice lead to different susceptibilities due to MHC polymorphisms and immune factors (Else and Wakelin, 1988). The H-2^k^ haplotype (BALB/k, B10.BR) mouse strains are more susceptible to *T. trichiura*, while H-2^b^ (B10) or H-2^q^ (B10.G) background strains are more resistant. Host sex also affects resistance to *T. trichiura via* a hormone-regulated immune response (Else and Wakelin, 1988). Dihydrotestosterone inhibits dendritic cell-mediated T-cell activation and suppresses Th2 responses through IL-8, and 17-β-estradiol enforces Th2 responses in an IL-4-dependent manner (Hepworth et al., 2010). Upon infection with *T. trichiura*, male IL-4-deficient BALB/c mice were found to exhibit chronic infection, while females displayed acute infection (Artis et al., 1999), suggesting that IL-4 plays a genetic-dependent role in the immune response of hosts induced by *T. trichiura*. In addition, another cytokine, IL-13, is reported to reduce worm burden (Hayes et al., 2007).

#### 2.6.2 Hookworms

A total of 9 species of zoonotic hookworms have been found, among which *Ancylostoma duodenale* and *Necator americanus* are the main infection sources to humans. Others, such as *Ancylostoma ceyloides (A. ceyloides)*, *Ancylostoma canis (A. canis)* and *Ancylostoma brasiliensis (A. brasiliensis)*, also infect humans under certain circumstances. Hookworms manifest obvious infection diversity in hosts (Seguel and Gottdenker, 2017). *Ancylostoma caninum*, for example, is believed to parasitize only dogs and has been found to infect humans and cats in some cases (Bahgat et al., 1999; Kwon et al., 2003; Liu et al., 2013). The climate is also closely associated with infection. Epidemiological investigation has revealed that the prevalence of hookworm infection is much lower in June and winter than in the monsoon season (Croese, 1995).

Humans act as nonpermissive hosts of *A. brasiliensis*, and worms fail to develop into adults. Mouse models are the most commonly used models to study the host adaptation of *A. brasiliensis*. Studies have noted the important roles of Th2 immune responses in the host adaptation of *A. brasiliensis*. Th2-type cytokines such as IL-4 and IL-13 limit reproduction and survival and promote the elimination of worms (Urban et al., 1995; Gerbe et al., 2016; Symowski and Voehringer, 2019). The phospholipase A2 group 1b (pla2g1b) excreted by host epithelial cells induces Th2 immune responses to kill *A. brasiliensis*, while mice lacking pla2g1b fail to expel worms (Entwistle et al., 2017). The expression of pla2g1b in epithelial cells relies on the intestinal microbiota, adaptive immunity and coenzyme γ chain-dependent signaling. Due to the differences in genetic backgrounds, pla2g1b expression is distinctive among hosts, which is speculated to affect the host adaptation of *A. brasiliensis* (Entwistle et al., 2017). In addition, the inflammation-related transcription factor STAT6 enhances worm elimination by promoting hookworm capture and degradation in host mucosa (Ishiwata et al., 2002; Van Panhuys et al., 2013).

#### 2.6.3 Strongyloides stercoralis

*Strongyloides stercoralis (S. stercoralis)* is a species of facultative parasites, and the life cycle includes free-living and parasitic generations. The hosts of parasitic generations comprise humans, dogs, cats, gerbils and mice (Lok, 2007; Bonne-Annee et al., 2011; Dantas-Torres and Otranto, 2014). *S. stercoralis* derived from humans can also infect dogs (Jaleta et al., 2017), resulting in chronic infection (Schad et al., 1984). Some hosts, including gerbils and C57BL/6J and SCID mice, show natural resistance to *S. stercoralis* infection by inhibiting the development of worms (Dawkins and Grove, 1982; Nolan et al., 1999).

The adults of *S. stercoralis* parasitize the small intestine of the host, and larvae can migrate into the main organs, such as the heart, liver, spleen and lungs, leading to strongyloidiasis. Before invasion, the larvae probably distinguish hosts and nonhosts by sensing host smell and skin temperature through the cGMP-gated cation channel subunit tax-4 (Bryant et al., 2018; Gang et al., 2020). In addition, the CO_2_ concentration and unique uridylic acid of skin also impact larval infection, but the molecular mechanism remains elusive (Safer et al., 2007). DAF-12 is a nematode-specific nuclear receptor and probably governs the development of *S. stercoralis* (Cheong et al., 2021), providing a therapeutic target for strongyloidiasis. As reported, treatment with Δ7-dafachronic acid significantly reduces worm burden by targeting DAF-12 (Patton et al., 2018). In terms of the host, the hormone level and immune factors exhibit effects on *S. stercoralis* infection. Recent studies have shown that endogenous cortisol mimics parasitic ecdysone and promotes the development of bacillary cercariae to filamentous cercariae, leading to persistent infection (Teixeira et al., 2016). Immunocompetent patients infected by *S. stercoralis* regularly present asymptomatic infections, while immunocompromised patients exhibit severe infections (Abanyie et al., 2015). The underlying mechanism might be impaired MHC II-mediated Th2 responses (Rodrigues et al., 2009).

#### 2.6.4 Trichinella spiralis

*Trichinella spiralis* (*T. spiralis*) is one of the most frequent parasites of carnivorous mammals (Pozio, 2000; Scandrett et al., 2018). The adult worms infest the duodenum and jejunum of diverse hosts, where they lay newborn larvae that reach skeletal muscle with blood circulation and form infectious larval sacs. The life cycle of *T. spiralis* can be completed in the same host; thus, the host serves as both an intermediate host and a definitive host (Pozio, 2000; Scandrett et al., 2018). In view of different genetic backgrounds, different hosts infected by *T. spiralis* present distinct phenotypes, including cystic and noncystic foci (Pozio et al., 2004), suggesting that genetic factors affect pathogenicity. The development of *T. spiralis* in hosts is vulnerable to microenvironmental changes caused by immune and nonimmune factors (Huang et al., 2015). Eosinophils can promote larval growth by inhibiting local inflammation and enhancing nutrient absorption and metabolism (Huang et al., 2015). In contrast, intestinal mast cells promote the exclusion of worms from the gastrointestinal tract by increasing the permeability of intestinal epithelial cells (McDermott et al., 2003). Leptin reduction is involved in facilitating parasite expulsion by augmenting protective Th2 immune responses (Worthington et al., 2013). However, β-glucan enhances worm expulsion through the mucus layer independent of Th2 immunity (Jin et al., 2022).

#### 2.6.5 Filaria

*Filaria* comprises multiple subspecies, of which nearly 8 infect humans, yet the parasitic sites and pathological outcomes vary greatly as a result of the genetic differences among hosts. Infected patients often present severe lymphadenitis or blindness. The hosts of filaria include intermediate and definitive hosts. The intermediate hosts of *Filaria* vary from mosquitoes, *Simulium*, and *Tabanus* to *Culicoides* (Brianti et al., 2012). Humans and some animals, such as dogs, foxes, wolves and cats, serve as definitive hosts (McNulty et al., 2013) and become infected when bitten by mosquitoes containing infective host-specific *Filaria* subspecies (Vakalis and Himonas, 1997). Generally, animal-specific *Filaria* fail to develop sexually and produce microfilariae in humans. This is likely attributed to the host immune response promoting worm elimination (Pampiglione et al., 1995).

In experimental animal models with distinct genetic backgrounds, the susceptibility and resistance to *Filari* also exhibit great differences. *Litomosoides sigmodontis* (*L. sigmodontis*) can develop into sexual maturity in BALB/c mice, and the infectious microfilariae can be released into the surrounding environment; however, in C57BL/6 mice, the worms are eliminated before microfilariae are released (Petit et al., 1992; Graham et al., 2005; Attout et al., 2008). One of the most likely causes is the stronger Th1/Th2 immune responses present in C57BL/6 mice (Babayan et al., 2003). In addition, C57BL/6 mice with MHC class-II and RAG2IL-2Rγ loss show impaired resistance to *Filaria*, suggesting that adaptive immunity plays a crucial role during *L. sigmodontis* infection (Hoffmann et al., 2001; Wiszniewsky et al., 2021). In line with these findings, cytokines (IL-4, IL-10, IL-17 and IFN-γ) and chemokines (CXCL12 and CCL17) tightly related to adaptive immunity have evident effects on the development, migration and survival of infective larvae (Hoffmann et al., 2001; Saeftel et al., 2001; Specht et al., 2011; Bouchery et al., 2012; Ritter et al., 2018). IL-4, IL-17, IFN-γ, and CXCL12 are required to repress filarial nematode development and survival, while IL-10 promotes the persistence of *L. sigmodontis* Mf in hosts; CCL17 controls filarial larval entry by inhibiting vascular permeability. Furthermore, the chemokine receptor CCR3 usually mediates the recruitment of innate immune cells, and BALB/c mice lacking CCR3 exhibit higher susceptibility to human-specific *Loa loa castellani*, indicating the critical role of innate immunity in the anti-infection abilities of *L. sigmodontis* (Fombad et al., 2019). Consistently, the accumulation of innate immune cell neutrophils in hosts significantly inhibits the motility and pathogenicity of *L. sigmodontis* (Frohberger et al., 2020). Moreover, *L. sigmodontis* was found to exploit coevolution with other pathogens, such as *Wolbachia*, to achieve immune escape and enforce host adaptation (Slatko et al., 2014; Lefoulon et al., 2016). The killing of *Wolbachia* with antibiotics favors *Filaria* elimination (Taylor et al., 2014), which is likely attributable to the perturbation of the host immune response by *Wolbachia* (Brattig, 2004).

#### 2.6.6 Angiostrongylus cantonensis

*Angiostrongylus cantonensis* (*A. cantonensis*) remains one of the leading causes of eosinophilic meningoencephalitis worldwide. Infections often occur through the ingestion of food or contaminated water containing third-stage larvae (Zhou et al., 2019). The definitive hosts of *A. cantonensis* include permissive hosts (rats, tree shrews, dogs, meerkats, tree rats, etc.) and nonpermissive hosts (mice, humans, parrots, Mongolian gerbils, etc.) (Wallace and Rosen, 1969; Reece et al., 2013; Okano et al., 2014; Wei et al., 2014; Zhou et al., 2019). In permissive hosts, the worms develop into fourth-/fifth-stage larvae in the host brain, resulting in meningeal thickening and a severe inflammatory response. However, at 28 days after infection, the worms migrate from the brain to the lung, where they develop into adults and lay eggs along with mitigated pathological responses in the brain (Zhou et al., 2019). In nonpermissive hosts, the worms fail to leave the lungs and die in the brain, leading to severe eosinophilic meningoencephalitis (Zhou et al., 2021).

Although it is generally acknowledged that *A. cantonensis* fails to move from the cerebrum to pulmonary tissue in nonpermissive hosts such as humans, parrots and *Mongolian* gerbils, sporadic cases with worms present in the lungs are still observed. In humans, the worms were observed in both the brain and lungs of a male infant infected with *A. cantonensis* (Lindo et al., 2004). In addition, the worms of *A. cantonensis* are also found in the lungs of parrots and *Mongolian gerbils* (Reece et al., 2013; Wei et al., 2014). Such examples reflect the fact that some key factors determine the host adaptation of *A. cantonensis*. In addition, the perturbation of these factors might affect the migration and development of worms in hosts. When fifth-stage larvae from the brains of the permissive host rats were transferred into the brains of the nonpermissive hosts rabbits or mice, no worms could migrate to the lungs (Ko, 1979), suggesting that the genetic backgrounds of hosts likely determine worm migration. In mice without thymuses or eosinophils, *A. cantonensis* larvae acquire the ability to migrate from the brain to the lungs but fail to develop sexually, indicating that larval migration in hosts occurs in an immunity-dependent manner, yet development relies on other factors (Yoshimura et al., 1982; Sasaki et al., 1993).

## 3 Conclusions

**Figure 1:**
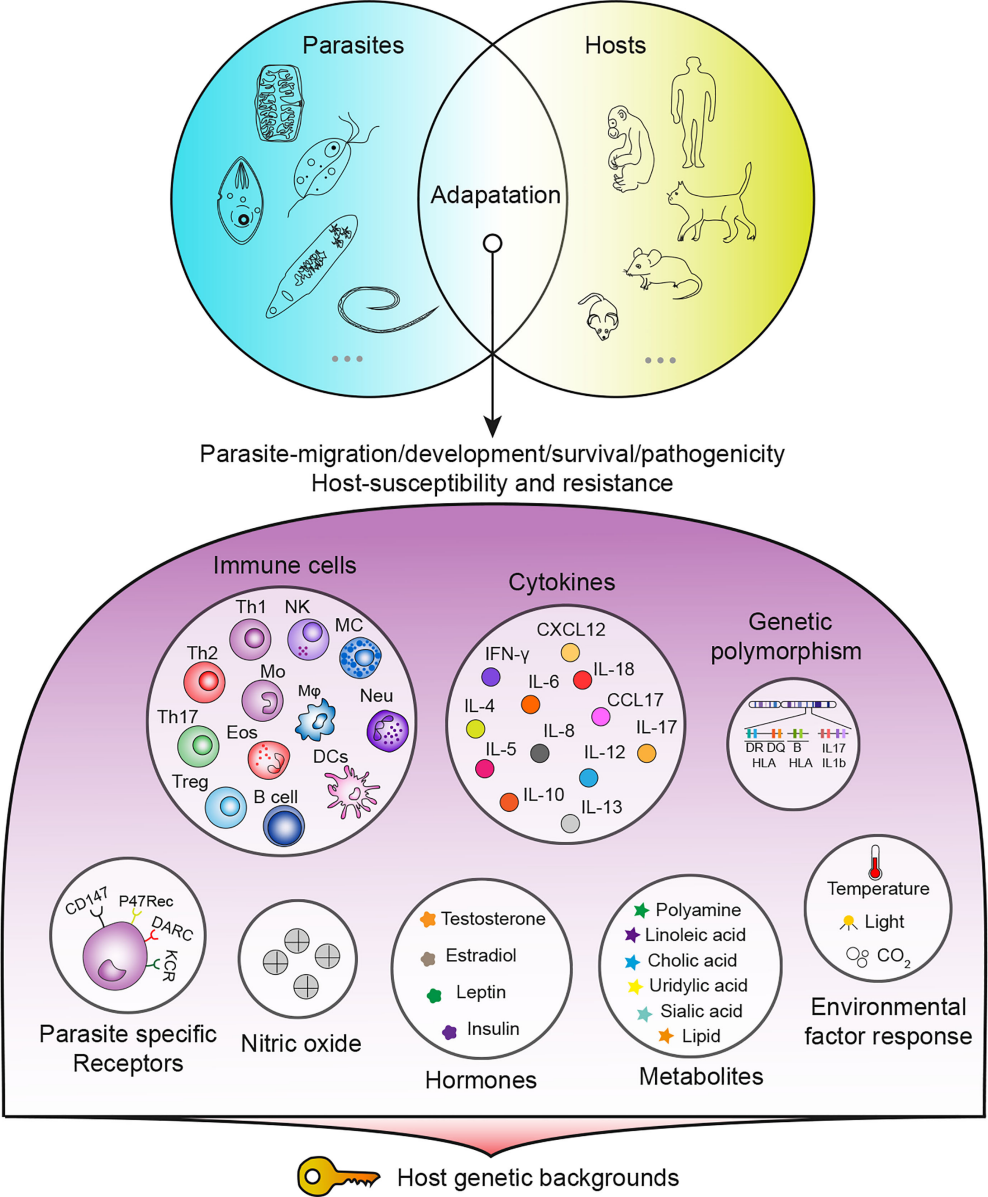
Summary of host genetic background-related factors affecting parasite-host adaptation.

**Figure 2:**
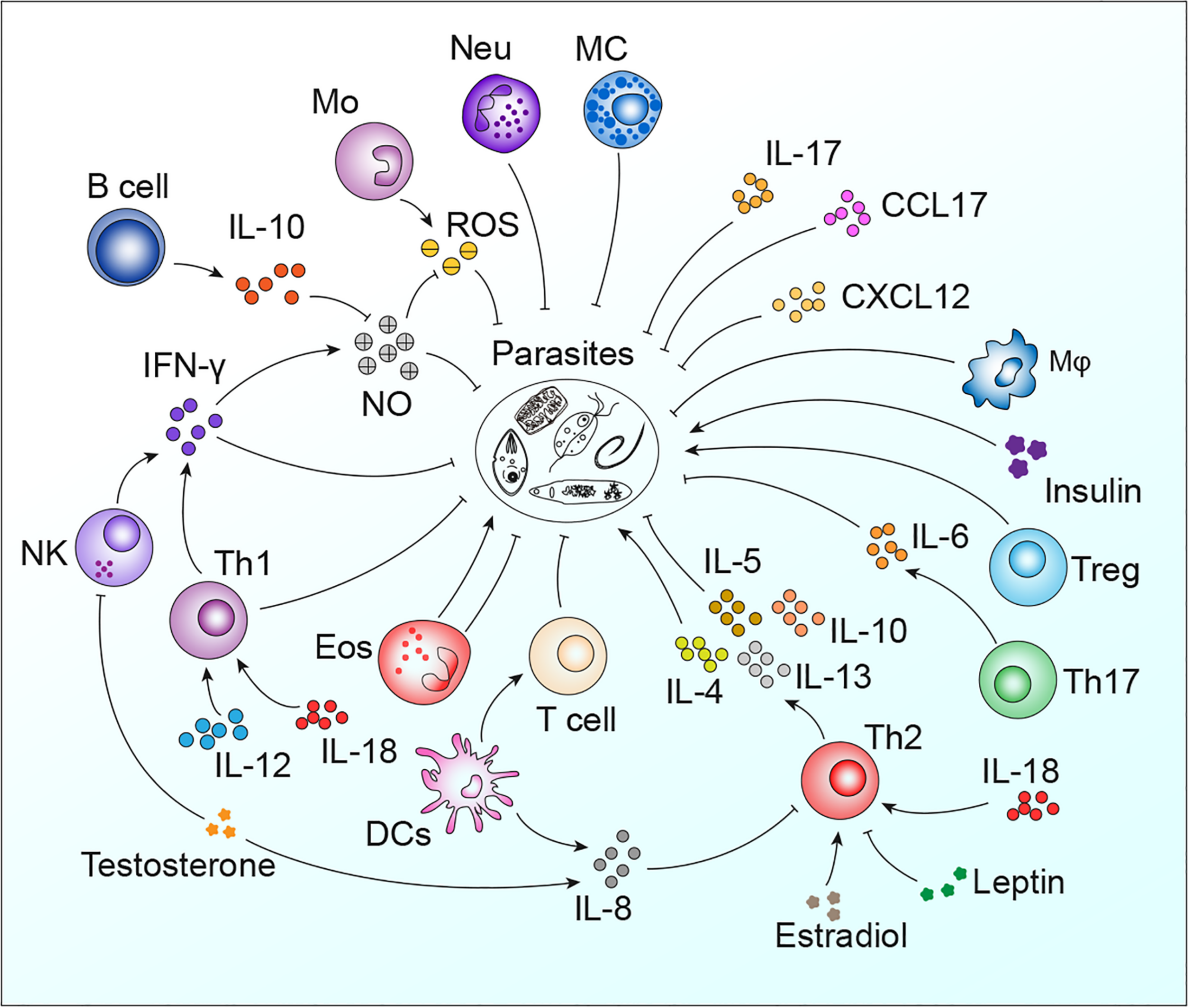
The interaction network of host genetic background- and immune-related factors involved in parasite resistance. Mφ, macrophages; Neu, neutrophils; Mo, monocytes; MC, mast cells.

Parasites remain one of the most threatening pathogens to humans worldwide. A promising strategy to control parasitosis is to intervene in deleterious parasite-host adaptation, which comprises parasite migration, development, survival and pathogenicity in hosts as well as host susceptibility and resistance to parasites. As a consequence of the distinct genetic backgrounds of hosts, after infection, parasite-host adaptations vary and result in different outcomes. In this article, parasite-host adaptation closely correlated with host genetic background-related factors and the underlying mechanisms were reviewed. In summary, various genetically related factors, including hormones, NO, immune cells, cytokines, genetic polymorphisms and metabolites, affect parasite-host adaptation (**Figure 1**), among which most factors function by modulating host immune responses. Thus, we have established an interaction network of host genetic background- and immune-related factors involved in parasite resistance to better depict the underlying mechanisms (**Figure 2**). Importantly, the host genetic background generally determines the differences in immune factors. The genetic background of the C57BL/6 mouse strain leads these mice to possess stronger Th1 response components and thus commonly a higher resistance to parasites than BALB/c mice. In contrast, the genetic background of the BALB/c mouse strain leads these mice to present a stronger Th2 response than C57BK/6 mice and often exhibit susceptibility to parasites. In addition, host genetic backgrounds also influence hormone levels, which could affect parasite resistance in immune-dependent or immune-independent ways (**Figure 2**; **Table 1**). The majority of immune-related factors exert explicit antiparasite (Th1, NK, B cells, DC, etc.) or proparasite (Treg and insulin) functions apart from NO, eosinophil, and Th2 response components. In hosts with distinct genetic backgrounds, a lack of NO is positively or negatively correlated with parasite resistance (**Table 2**), eosinophils play dual roles in the expulsion and development of parasites, and Th2 cells and related cytokines exhibit contradictory functions (**Tables 3**, **4**). For nonpermissive hosts, the Th2 response regularly promotes parasite elimination, while the Th2 response is beneficial for parasite survival (**Tables 3**, **4**). Furthermore, genetic polymorphisms (mainly MHC polymorphisms) and serum immunoglobulin and complement levels display obvious correlations with host susceptibility and resistance (**Table 5**). Excitingly, sera derived from patients with parasitosis have shown diagnostic and therapeutic potential. In addition, the existence of parasite-specific receptors on host cells confers infection susceptibility, host-derived metabolites can favor the survival and development of larvae, and some hosts display responses to environmentally induced factors to affect susceptibility (**Table 6**).

**Table 1:** Role of hormone in parasite-host adaptation.

Hormone	Parasite	Primary Activity	Ref
Testosterone	*E. histolytica*	Increases susceptibility by inhibiting IFN-γ secretion of natural killer T cells	(Lotter et al., 2013)
	*S. mansoni*	Enhances the resistance and reduce the worm burden	(Nakazawa et al., 1997)
	*T. trichiura*	Inhibits host resistance by suppressing T cells activation and Th2 response through IL-8	(Hepworth et al., 2010)
Estradiol	*T. trichiura*	Enforces host resistance by enhancing Th2 response in an IL-4 dependent manner	(Hepworth et al., 2010)
	*T. vaginalis*	Helps the adhesion of T. vaginalis	(Martinotti et al., 1988)
Leptin	*E. histolytica*	Increase resistance to *E. histolytica* infection	(Duggal et al., 2011)
	*T. spiralis*	Facilitating *T. spiralis* excretion by negatively regulating protective Th2 immune response	(Worthington et al., 2013)
Insulin	*E. multilocularis*	Promote the development of *E. multilocularis* through evolutionarily conserved insulin signaling pathway of worms	(Hemer et al., 2014)

**Table 2:** Role of NO in parasite-host adaptation.

Parasite	Mechanism	Function	Ref
*E. histolytica*		+	(Ivory et al., 2007; Ramirez-Emiliano et al., 2007)
*T. cruzi*	By downregulating IL-10	+	(da Rocha et al., 2019)
*S. japonicum*	Inhibits the development of *S. japonicum*	+	(Shen et al., 2017)
*hymenolepis*		+	(Mahmoud and Habib, 2003)
*E. granulosus*		+	(Mourglia-Ettlin et al., 2016b, Amri et al., 2009)
*E. multilocularis*		–	(Dai et al., 2003)
*T. gondii*		+	(Witola et al., 2017)
*T. gondii*	NO deficiency trigger ROS production of the mononuclear phagocyte system	–	(Wang et al., 2021)

+, heighten host resistance.

- , heighten host susceptibility.

**Table 3:** Role of cytokines and chemokines in parasite-host adaptation.

Cytokines chemokines	Parasite	Primary Activity	Ref
IFN-γ	*E. histolytica*	Induces NO production and promotes the elimination of amoeba	(Duggal et al., 2011; Lotter et al., 2013)
	*C. sinensis*	Higher levels of IFN-γ and Th1 immune responses shows lower susceptibility of host to C. sinensis infection	(Chessler et al., 2011)
	*L. sigmodontis*	Limits filarial nematode development of *L. sigmodontis* Mf in host	(Saeftel et al., 2001)
	*T. gondii*	Induces IL-12 to drive the Th1 immune response and enhance host resistance to *T. gondii*.	(Dupont et al., 2012)
IL-12 and IFN-γ	*T. gondii*	IL-12-induced IFN-γ production enhances host resistance to *T. gondii*	(Dupont et al., 2012)
IL-4	*L. sigmodontis*	Limit filarial nematode development and survival	(Hoffmann et al., 2001; Ritter et al., 2018)
IL-4 and Il-10	*E. granulosus*	IL-4 and Il-10 display opposite killing roles to *E. granulosus* by elevating the levels of NO	(Mourglia-Ettlin et al., 2016b, Amri et al., 2009)
IL-4 and IL-13	*S. mansoni*	IL-4 and IL-13 is beneficial for the parasite in the permissive host while in the non-permissive host, they seem to play a host-protective role	(Khalife et al., 2000)
	*A. brasiliensis*	Limit the reproduction, survival and promote the elimination of *A. brasiliensis* in mouse	(Urban et al., 1995; Gerbe et al., 2016; Symowski and Voehringer, 2019)
IL-5 and IL-13	*H. diminuta*	Promote the expulsion of *H. diminuta*	(McKay and Khan, 2003; Ryan and Oghumu, 2019)
IL-6	*E. histolytica*	IL-6 promotes anti *E. histolytica* response by Th17 cells	(Groneberg et al., 2022)
IL-8	*T. trichiura*	IL-8 enhances host resistance to *T. trichiura*	(Hepworth et al., 2010)
IL-10	*L. sigmodontis*	Promotes the persistence of *L. sigmodontis* Mf in host	(Hoffmann et al., 2001; Ritter et al., 2018)
	*E. histolytica*	IL-10 KO mouse exhibits susceptibility of host to amebic invasion by defective synthesis of mucin that could cleave the amoebic caspase	(Ivory et al., 2007)
	*T. cruzi*	IL-10 suppresses NO production and enhances host susceptibility to *T. cruzi*	(da Rocha et al., 2019)
IL-13	*T. trichiura*	Reduce *T. trichiura* burden of host	(Hayes et al., 2007)
CXCL12and IL-17	*L. sigmodontis*	Limit filarial nematode development and survival of *L. sigmodontis* Mf in host	(Bouchery et al., 2012; Ritter et al., 2018)
IL-18	*Leishimania*	IL-18 can be either host-protective or disease-exacerbative during leishimania infections, depending on the genetic background determined balance of Th1/Th2 responses	(Wei et al., 2004)
CCL17	*Filaria*	CCL17 controls filarial larval entry by inhibiting vascular permeability	(Specht et al., 2011)

**Table 4:** Role of immune cells in parasite-host adaptation.

Immune cells	Parasite	Primary Activity	Ref
B-1 cells	*T. cruzi*	Secreting excessive IL-10 and reduces NO, leading to higher susceptibility of BALB/c mice to *T. cruzi*	(da Rocha et al., 2019)
Pre-T cells	*T. vaginalis*	Enhance stronger resistance of host to *T. vaginalis*	(Martinotti et al., 1988)
T cells	*T. vaginalis*	The sensitivity of T cells and activation of phagocytes in host determine the resistance to primary infection with T. vaginalis by regulating Th1/Th2 response	(Caterina et al., 1996)
	*Cryptosporidium*	Mediate adaptive immunity and impairs host adaptation of *Cryptosporidium*	(Winfield, 1997)
	*E. granulosus*	T cell-mediated stronger immune responses to *E. granulosus* in the host	(Garcia-Luna et al., 2021; Miles et al., 2020)
Th1 cells	*Leishmania*	Heighten host resistance to *Leishmania* by secreting Th1-type cytokines	(Wei et al., 2004)
	*T. gondii*	Producing IL-12-induced IFN-γ enhances host resistance to *T. gondii*	(Dupont et al., 2012)
	*F. hepatica*	Promotes the elimination of host to *F. hepatica*	(Pleasance et al., 2011)
	*E. granulosus*	Protect against hydatid cyst of *E. granulosus*	(Garcia-Luna et al., 2021; Miles et al., 2020)
Th2 cells	*E. granulosus*	Dual functions to parasite depending on the genetic backgrounds of host	(Garcia-Luna et al., 2021; Miles et al., 2020)
	*S. mansoni*	Beneficial for the *S. mansoni* in permissive host while in the non-permissive host, they seem to play a host-protective role	(Khalife et al., 2000)
	*A. brasiliensis*	Limit the reproduction, survival and promote the elimination of parasite by secretion of IL-4 and IL-13	(Urban et al., 1995; Symowski and Voehringer, 2019; Gerbe et al., 2016)
	*S. stercoralis*	Elevate the resistance of host to *S. stercoralis*	(Rodrigues et al., 2009)
	*T. spiralis*	Enhance the expulsion of *T. spiralis*	(Worthington et al., 2013)
Th17 cells	*E. histolytica*	Th17 cells regulated by HIF-1α determine the difference of immunopathology between male and female	(Groneberg et al., 2022)
Mast cells	*H. nana*	Mast cells enforce the resistance of host	(Gonzalez et al., 2018)
Mast cells	*T. spiralis*	Promote the exclusion of worms by increasing Permeability of intestinal epithelial cells	(McDermott et al., 2003)
NK cells	*E. histolytica*	Enhance host resistance through secreting IFN-γ which is regulated by host hormone levels	(Lotter et al., 2013; Duggal et al., 2011)
Mononuclear phagocytes	*T. gondii*	Releasing ROS to enforce host resistance to *T. gondii*	(Wang et al., 2021)
Kupffer cells	*E. granulosus*	Help hydatid cyst of *E. granulosus* to parasitize host liver through the specific receptor KCR	(Fadden et al., 2003)
Eosinophils	*S. mansoni*	Eliminate worm	(Shen et al., 2020)
	*A. cantonensis*	Alter the migration route of larvae from brain to lung but fail to develop sexually mature	(Yoshimura et al., 1982; Sasaki et al., 1993)
	*T. spiralis*	Promote larvae growth by inhibiting local inflammation and enhancing nutrient absorption and metabolism	(Huang et al., 2015)
Macrophages	*S. mansoni*	Eliminate worm	(Shen et al., 2020)
Neutrophils	*E. multilocularis*	Lower the susceptibility of host to *E. multilocularis*	(Joekel et al., 2021)
	*H. nana*	enforce the resistance of host	(Gonzalez et al., 2018)
	*L. sigmodontis*	Inhibit the motility and pathogenicity of *L. sigmodontis*	(Frohberger et al., 2020)
Dendritic cells	*T. trichiura*	Mediate T cells activation and suppress Th2 cells through IL-8, resulting in stronger resistance to *T. trichiura*	(Hepworth et al., 2010)

**Table 5:** Role of MHC polymorphisms, humoral immunity and complement in parasite-host adaptation.

Categories	Parasite	Primary Activity	Ref
MHC polymorphisms	*E. histolytica*	HLA-DRB1*03:0101, HLA-DRB1*11:0101 in the humans are associated with higher susceptibility to amebiasis	(Al-Khaliq and Mahdi, 2018)
	*T. vaginalis*	Mouse strain harboring H-2d is susceptible to infection while strains with H-2k resistant	(Caterina et al., 1996)
	*E. granulosus*	HLA-DR3+ and HLA-DQ2+ haplotype is more susceptible to *E. granulosus*	(Dai et al., 2003)
	*T. trichiura*	H-2k haplotype mouse strains are more susceptible while H-2b or H-2q background strains are more resistant	(Else and Wakelin, 1988)
	*Cryptosporidium*	HLA DQB1*0301 and HLA B*15 are related to higher *Cryptosporidium* infection ratio	(Hunter et al., 2004)
Humoral immunity	*T. vaginalis*	The elevation of IgM, IgG1 and IgG2b levels are related to more severe symptoms caused by *T. vaginalis*	(Imam et al., 2007)
	*C. sinensis*	IgE lower the susceptibility of C3H/HeN mouse strain to *C. sinensis*	(Uddin et al., 2012)
	*S. japonicum*	IgG3 partially determines the resistance of *M. fortis* to *S. japonicum*, while that of rat depends on IgG2a and IgE	(Shen et al., 2020; Li et al., 2013; Jiang et al., 2010)
	*E. granulosus*	IgG1 in C57BL/6 serum shows explicit therapeutic efficiency to BALB/c mice infected by *E. granulosus*	(Mourglia-Ettlin et al., 2016)
Complement levels	*E. histolytica*	The serum complements promote elimination of amebiasis at early infection stage	(Olivos-Garcia et al., 2018)

**Table 6:** Role of other factors in parasite-host adaptation.

Categories	Parasite	Primary Activity	Ref
Parasite-specific receptors	*Pf*	Rh5 molecule of *Pf* is the only ligand present in all Pf strains that could bind to basigin (BSG, CD147) on human erythrocytes surface resulting in the specific infection of *Pf* to humans	(Crosnier et al., 2011; Forni et al., 2015)
	*Pf*	Pfs47 on the surface of *Pf* binding to P47Rec specifically expressed in the stomach of Anopheles mosquitoes mediates the immune escape of ookinete	(Molina-Cruz et al., 2020)
	*Pv*	DBP-RII on the surface of *Pv* and DARC on the surface of erythrocytes is an essential molecular basis for the infection of host	(Payne et al., 2017; de Sousa et al., 2014; Chitnis and Sharma, 2008)
	*E. granulosus*	KCR on the surface of kuffer cells attributes for most common liver involvement of host upon infection with *E. granulosus* as a result of binding to hydatid cyst laminated layer glycans	(Diaz et al., 2015; Fadden et al., 2003)
Metabolites	*T. cruzi*	Polyamine promotes *T. cruzi* replication	(Abad Dar and Holscher, 2018)
	*T. gondii*	Linoleic acid is the raw material for the development of sexual stage of *T. gondii*	(Martorelli Di Genova et al., 2019)
	*S. japonicum*	Lipid promotes the development of *S. japonicum*	(Shen et al., 2017)
	*C. sinensis*	The chemotaxis of Cholic acid chemotaxis to *C. sinensis*	(Li et al., 2018)
	*S. stercoralis*	The chemotaxis of uridylic acid to *S. stercoralis*	(Safer et al., 2007)
	*Plasmodium*	Sialic acid affects the affinity of *Plasmodium* to host erythrocytes	(Hayakawa et al., 2001; Muchmore et al., 1998)
Environmental factors	*S. mansoni*	Temperature affects host HSP 70 and HSP 90 expression, leading to the stronger susceptibility of BS-90 strains to *S. mansoni*	(Ittiprasert and Knight, 2012)
	*S. mansoni*	The light could modulate the susceptibility of *B. glabrata* to *S. mansoni*	(Steinauer and Bonner, 2012)
	*S. stercoralis*	Temperature and CO2 concentration of host skin can influence the infection of *S. stercoralis* to host through the cGMP-gated cation channel subunit tax-4	(Safer et al., 2007)

To further explore the impact of parasite-specific receptors and host metabolites on parasite adaptation, in future studies, a combination of molecular and biochemical approaches can be used. CRISPR/Cas9 technology can be used to knock out or overexpress specific host receptors and investigate their impact on parasitic infection. Metabolomics can also be employed to identify host-derived metabolites that affect parasite growth and development. Furthermore, the utilization of patient-derived serum for the diagnosis and treatment of parasitic diseases can be a promising strategy. Investigating the specific antibodies and immune molecules present in patient serum that contribute to parasite resistance can aid in the development of targeted therapies for parasitic diseases.

To further understand the complex mechanisms underlying parasite adaptation, in future studies, a multidisciplinary approach, integrating genetics, immunology, biochemistry, and ecology, can be employed. Combining genetic studies with ecological studies can provide insights into how host-parasite coevolution shapes the genetic diversity of both hosts and parasites. In addition, the integration of immunological and biochemical studies can elucidate the molecular mechanisms underlying the impact of host immune cells and molecules on parasite adaptation. Furthermore, the study of the impact of parasite adaptation on host physiological and behavioral traits can provide insights into the broader ecological consequences of parasitic infections. Investigations on the impact of parasite adaptation on host microbiota can also provide insights into the complex interactions among parasites, hosts and the microbiome.

In summary, through an understanding of the underlying mechanisms of parasite-host adaptation from the perspective of host genetic backgrounds, it is expected that promising candidate targets to block the survival and development as well as the pathogenicity of parasites will be obtained. Interventions targeting key factors involved in host adaptation, enhancing the antiparasite immune response, exploiting patient-derived serum, blocking parasite-specific receptors, or interfering with the development and survival of parasites through inhibitors and diet restrictions, could ultimately achieve further advances in parasitosis control and therapy and deepen the understanding of the adaptive differences among parasites in different hosts.

## Author contributions

HZ and JL conceived this study and revised the manuscript. CY, LZ, LT, and YD undertook the literature review and drafted the manuscript. All authors contributed to the article and approved the submitted version.

## Glossary

**Table:**

A. brasiliensis	Ancylostoma brasiliensis
A. cantonensis	Angiostrongylus cantonensis
C. hominis	Cryptosporidium. hominis
C. parvum	Cryptosporidium. parvum
C. sinensis	Clonorchis sinensis
DARC	Duffy antigen receptor chemokine
DBP-RII	Duffy-binding protein
E. granulosus	Echinococcus granulosus
E. histolytica	Entamoeba histolytica
E. multilocularis	Echinococcus multilocularis
F. hepatica	Fasciola hepatica
H. diminuta	Hymenolepis diminuta
H. nana	Hymenolepis nana
iNOS	inducible nitric oxide synthase
KCR	Kupffer cell-specific receptor
KO	knockout
L. sigmodontis	Litomosoides sigmodontis
L. braziliensis	Leishmania braziliensis
MHC	major histocompatibility complex
mMCP-1	mast cell-specific protease
NO	nitric oxide
Pf	Plasmodium. falciparum
pla2g1b	phospholipase A 2 group 1b
Pk	Plasmodium. knowlesi
Pv	Plasmodium. vivax
Pm	Plasmodium. malariae
Po	Plasmodium. ovale
S. haematobium	Schistosoma haematobium
S. japonicum	Schistosoma japonicum
S. mansoni	Schistosoma mansoni
S. malayensis	Schistosoma malayensis
S. mekongi	Schistosoma mekongi
SNP	single nucleotide polymorphism
S. intercalatum	Schistosoma intercalatum
T. b. gambiense	Trypanosoma brucei gambiense
T. b. rhodesiense	Trypanosoma brucei rhodesiense
T. cruzi	Trypanosoma cruzi
T. gondii	Toxoplasma gondii
T. spiralis	Trichinella spiralis
T. trichiura	Trichuris trichiura
T. vaginalis	Trichomonas vaginal
